# Endobronchial Inflammatory Myofibroblastic Tumor in a 3-Year-Old Child

**DOI:** 10.1055/a-2021-8054

**Published:** 2023-03-16

**Authors:** Riccardo Guanà, Andrea Carpino, Marta Miglietta, Elisa Zambaiti, Alessia Cerrina, Luca Lonati, Francesco Guerrera, Stefano Vallero, Salvatore Garofalo, Marco Bardessono, Francesca Maletta, Steffi Shilly, Fabrizio Gennari

**Affiliations:** 1 Division of Pediatric General, Thoracic & Minimally Invasive Surgery, University Hospital of Health and Science, Turin University, Regina Margherita Children's Hospital, Torino, Italy; 2 Columbia University School of Nursing, New York, New York, United States

**Keywords:** myofibroblastic tumor, bronchoscopy, thoracotomy

## Abstract

Inflammatory myofibroblastic tumor (IMT) is a mesenchymal tumor that can occur at any age. However, it is primarily seen in children, with the most common site being in the lung parenchyma, usually present with rare endobronchial lesions. This case reports the incidence in a 3-year-old girl diagnosed with pericardiac pneumonia treated with antibiotics with no clinical improvement. A chest computed tomography (CT) scan identified a 1.5-cm lesion in the left main bronchus. Bronchoscopy revealed complete obstruction of the left main stem bronchus. A left posterolateral thoracotomy was performed. Additionally, a left sleeve upper bronchial resection was conducted under fibroendoscopic control. Definitive histology confirmed IMT. After 2 years of endoscopic follow-up, there is no evidence of recurrence.

## Introduction

Inflammatory myofibroblastic tumor (IMT) is a mesenchymal tumor that can occur at any age but is predominantly seen in children and adolescents. 1 IMTs are a subset of inflammatory pseudotumors composed of myofibroblastic spindle cells accompanied by inflammatory infiltration of plasma cells, lymphocytes, and eosinophils. IMTs may be benign, malignant, recurrent, or metastatic. Their pathogenesis involves genetic mutation or they occur secondary to infections or autoimmune diseases. The most common site in young patients is lung parenchyma, where rare endobronchial lesions can be observed. The standard therapy for IMTs is complete surgical resection of the mass. We report the diagnostic work-up and subsequent surgical treatment and management of an endobronchial IMT in a small child.

## Case Presentation

A healthy 3-year-old girl was referred to our hospital for further investigation of pericardiac pneumonia. She was treated with oral antibiotics without any clinical improvement. A tuberculin skin test and bacteriological exam were initially conducted and were negative.

**Fig. 1:**
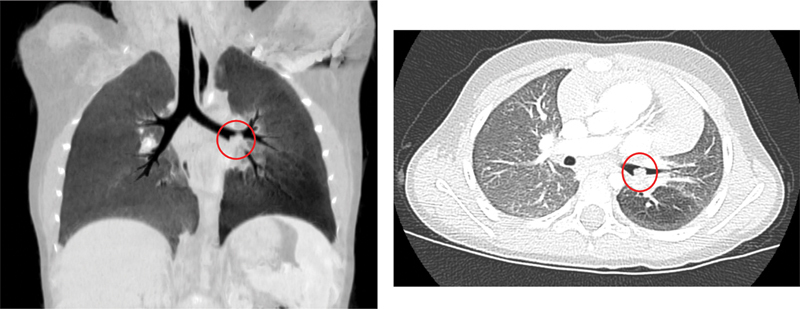
CT scan images of IMT.

**Fig. 2:**
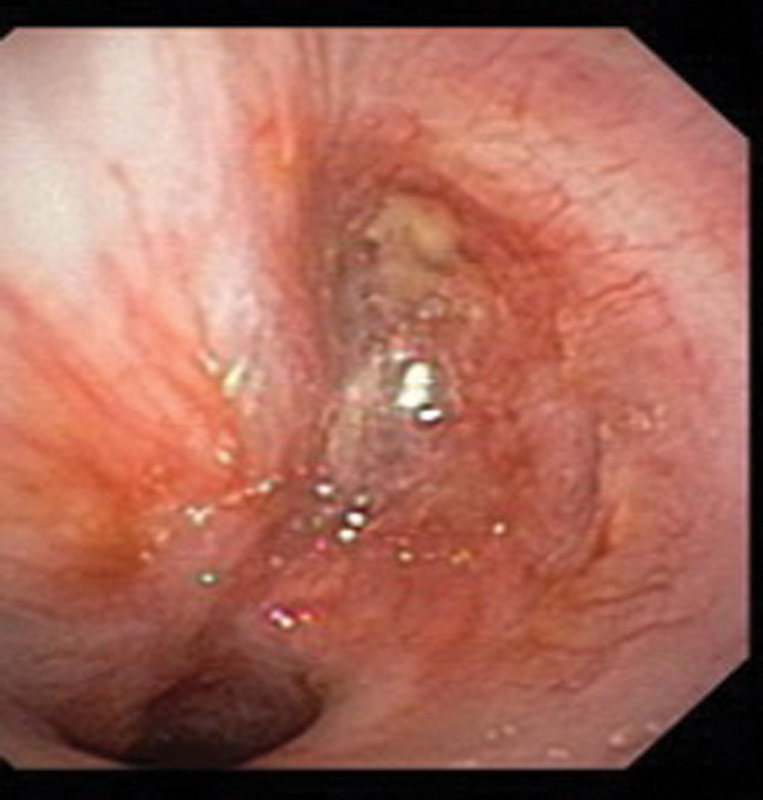
Endoscopic appearance of the endoluminal lesion in the left main bronchus, obliterating almost all the bronchial lumen.

After 2 months, in the clinical doubt of active pulmonary tuberculosis, a chest computed tomography (CT) scan was performed to exclude areas of consolidation or cavitation. Chest CT scan showed a peripheral atelectasis area in the left upper lobe and identified a 1.5-cm lesion in the left main bronchus ( Fig. 1 ). The fluorodeoxyglucose positron emission tomography–CT (PET/CT) scan indicated increased metabolism (maximum standardized uptake value [SUV], 3.5) without any other abnormal uptake in the body. Ten days after, the patient underwent flexible bronchoscopy, revealing the complete obstruction of the left main stem bronchus, which was attributed to a translucent and easily bleeding mass ( Fig. 2 ). Biopsies were obtained and showed small aggregates of spindle cells and inflammatory cells. Immunohistochemically, the spindle cells were positive for CD99, CD33 (focally), CD68 pgm1, and negative for chromogranin A, synaptophysin, cytokeratin AE1/AE3, and desmin. The K _i_ -67 proliferative index was low (2%). Immunohistochemistry was positive for ALK rearrangement, consistent with a possible diagnosis of IMT.

**Fig. 3:**
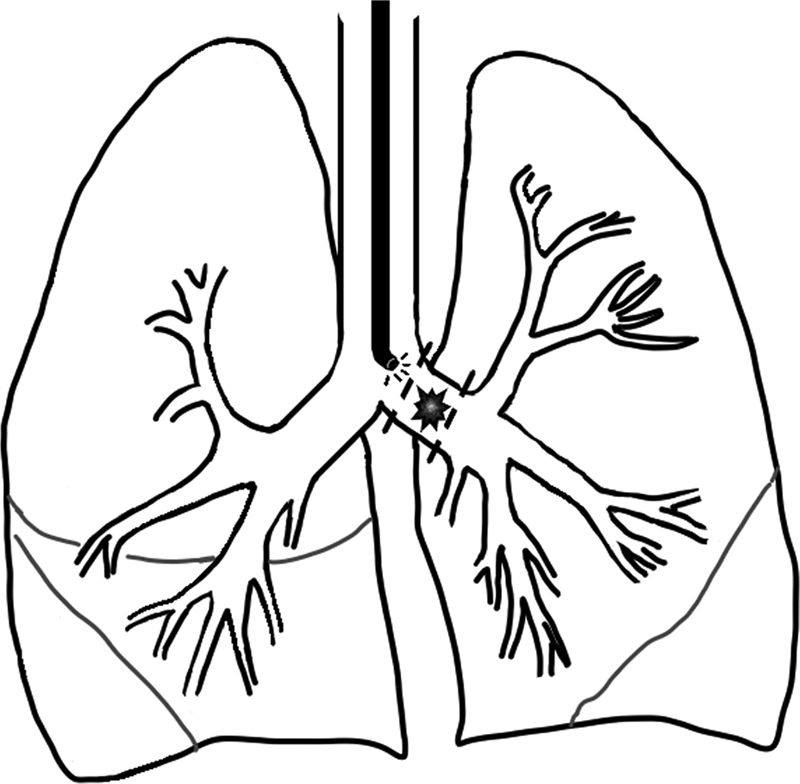
Schematic drawing of the surgical resection.

**Fig. 4:**
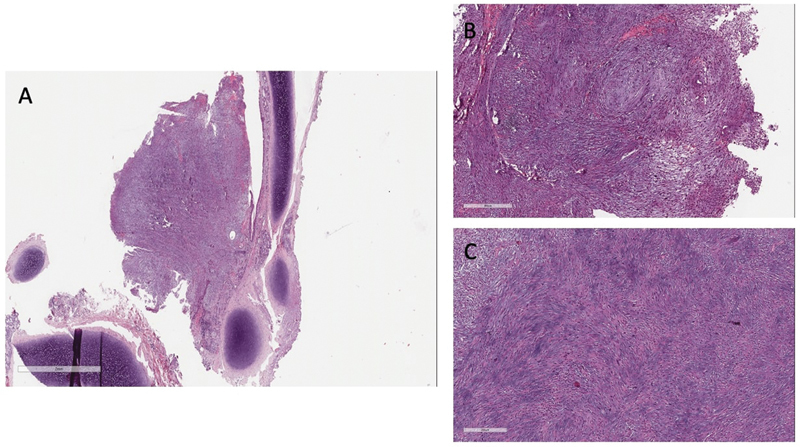
The endobronchial lesion is seen at low magnification (20 × ) as a polypoid mass protruding in the bronchial lumen ( **A** ); the lesion has a moderate cellularity with a fibrohistiocytic appearance composed by spindle cells (myofibroblasts) and a variable numbers of plasma cells, lymphocytes, histiocytes admixed; the stroma is focally myxoid (magnification 200 × ) ( **B, C** ).

Four months after the endoscopy, indication was given to remove the lesion surgically. The patient's weight at surgery was 15 kg. Fiberoptic intubation was used to achieve single-lung ventilation. A left posterolateral muscle-sparing thoracotomy was performed via the fifth intercostal space. The mass was identified by fibroendoscopy with transillumination of the proximal margin ( Fig. 3 ). All of the area was surrounded by postinflammatory scar tissue. Multiple lymph nodes at the mediastinal hilum were sampled, and the definitive histology was negative. A left sleeve upper bronchial resection was performed, preserving all the left-sided lung tissue. Additionally, an anastomosis with interrupted absorbable 5–0 sutures was achieved. The lesion appeared well circumscribed and was removed with macroscopic free margins without signs of infiltration. Two chest drains were left in place and removed on the third postoperative day. Definitive histology confirmed the initial diagnosis of IMT with free margins of resection ( Fig. 4 ).

There were no postoperative complications. The microscopic findings confirmed the IMT with free margins of resection. No recurrence was present at the radiologic and bronchoscopic follow-up 2 years postdiagnosis. The oncologist suggested clinical evaluation by a pulmonologist every 6 months in addition to chest X-rays alternating with CT scans every 6 months for the first 5 years.

## Discussion

The World Health Organization defines IMT as a myofibroblastic spindle cell population accompanied by an inflammatory infiltrate of plasma cells, lymphocytes, and eosinophils. Primary pulmonary tumors are rare in childhood. 1 Its etiology is not clearly defined, yet it has been postulated to be the consequence of an excessive response to tissue damage or a genetic reorganization of chromosome 23. Although it is thought to be asymptomatic, some patients develop cough, fever, chronic bronchitis, hemoptysis, chest pain, and dyspnea, among many other manifestations. In some instances, it can mimic pneumonia unresponsive to antibiotics, as seen in this case. Curtis et al 2 suggested that any child with persistent pneumonia that fails to resolve within 2 weeks of antibiotics should be referred for a CT scan and an early bronchoscopy. Generally, the radiologic findings are undefined. In our case, we performed a PET/CT to determine the TNM stage and to screen for metastases that might not be identified by CT alone. PET/magnetic resonance imaging (MRI) could be an alternative exam to avoid radiation exposure. In oncology, PET/MRI is feasible to perform equally as well as PET/CT; however, there is limited evidence. Studies including more patients and tumors are needed to establish PET/MRI indications and to identify appropriate protocols for each disease.

The most common presentation of IMT is a solitary, peripheral, well-circumscribed lung lesion. An endobronchial mass is rarely reported. Bronchoscopic examination with biopsy is the preferred method for diagnosing bronchial obstruction. IMT is considered a benign tumor; however, the recurrence rate is said to be 5% _._ Recurrence is more common in cases with incomplete lesion removal. Although IMT treatment is controversial, surgical resection is valuable for prognosis. The curative potentials of various bronchoscopic therapies, such as electric snare, carbon dioxide freezing, argon plasma coagulation, neodymium-doped yttrium aluminum garnet, laser, and photodynamic therapy, are often used in adult patients who have an intraluminal bronchial tumor. However, little knowledge exists about the effectiveness of these therapies for small children. Medical treatments, such as chemotherapy, corticosteroids, and nonsteroidal anti-inflammatory drugs, are used when surgery is contraindicated or when the tumor is metastatic.

The anaplastic lymphoma kinase (ALK) oncogene rearrangements have been documented in approximately 50% of IMTs and present as a subset that may be sensitive to targeted kinase inhibitors, such as crizotinib or ceritinib. 3 ALK rearrangements are neither exclusive nor specific to IMTs, as they can be found in other pediatric tumors (e.g., neuroblastoma, non-Hodgkin's lymphoma). Nonetheless, in this case, the patient possessed radiological, clinical, and histological features that suggested IMT among the possible differential diagnoses. ALK-positive immunostaining of the initial biopsy helped define an unspecific pathological picture.

Surgical excision may be performed with open thoracotomy, bronchoscopy, or video-assisted thoracoscopy. Lobectomy tends to be the most frequent procedure. Recently, lung-sparing techniques have rapidly increased. Given the lesion's location, the only surgical alternative may be a pneumonectomy. In our situation, we used a sleeve bronchial resection. While this procedure is employed in adult patients as both safe and effective for benign or low-grade malignant tumors, 4 it is less utilized in children whose structures are smaller and more delicate. Bronchial sleeve resection is ideal for frozen section to guarantee free margins and avoid resurgery. In our case, the lesions were well circumscribed and removed with macroscopic free margins without signs of infiltration. Frozen section would have warranted a more radical surgery.

Gaissert et al performed this procedure on 12 patients aged 8 to 19 years, demonstrating its safety (see Table 1 ). 5 Erginel et al presented one of the first cases of bronchial resection with a lung-sparing technique in patients under 6 years of age with endobronchial carcinoid. The case was described as an appropriate procedure for pediatric patients with bronchial tumors. 6 Pan et al reported one of the youngest cases of IMT of the left main bronchus treated by sleeve resection, considering it a viable alternative to pneumonectomy. 7 Different studies demonstrated that, when feasible, sleeve resection has a better outcome compared with pneumonectomy in terms of quality of life, operative mortality, and survival. 8 However, these series include mostly adults or adolescents. Additionally, none of the studies concern only IMT. To the best of our knowledge, this is one of the few cases of endobronchial IMT treated with sleeve resection in patients under the age of 5 years. Even if there are few accounts of this treatment in infants and younger children with endobronchial lesions, sleeve resection should be considered a feasible technique for younger patients and performed in high-specialized centers.

**Table 1:** Surgical approaches for endobronchial tumors in the literature

Authors	Year of publication	No. of patients	Age	Technique	Post-op complications	Follow-up time
Gaissert et al 5	1994	12	8–19 y	Bronchial resection	• One death • Prolonged atelectasis in three patients • Residual malacia in one patient	63 mo (range: 7–130 mo)
Erginel et al 6	2016	2	<6 y	Bronchoplastic techniques with preservation of the lung parenchyma	None	24 and 48 mo, respectively
Pan et al 7	2014	1	4 y	Left lower sleeve lobectomy	None	16 mo

## Conclusions

Endobronchial IMT is a rare mesenchymal tumor that can occur in children and adolescents. It should be considered a possible diagnosis in any patient who presents with intractable cases of pneumonia or lung consolidations that are unresponsive after medical treatment. In the reported cases, surgical resection appeared to be most effective in preventing recurrence.

## Notes

New Insights and the Importance for a Pediatric Surgeon Inflammatory myofibroblastic tumor (IMT) is a mesenchymal tumor that can occur at any age, but it is predominant in children and adolescents, usually present with rare endobronchial lesions. In case of bronchial masses or persistent pneumonia, IMT should be considered.

## References

[1] Karnak I Senocak M E Ciftci A O Inflammatory myofibroblastic tumor in children: diagnosis and treatment J Pediatr Surg 2001 36 06 908 912 11381424 10.1053/jpsu.2001.23970

[2] Curtis JM Lacey D Smyth R Carty H Endobronchial tumours in childhood Eur J Radiol 1998 29 01 11 20 9934553 10.1016/s0720-048x(97)00185-x

[3] Brivio E Zwaan C M ALK inhibition in two emblematic cases of pediatric inflammatory myofibroblastic tumor: efficacy and side effects Pediatr Blood Cancer 2019 66 05 e27645 30697903 10.1002/pbc.27645

[4] Yavuzer S Yüksel C Kutlay H Segmental bronchial sleeve resection: preserving all lung parenchyma for benign/low-grade neoplasms Ann Thorac Surg 2010 89 06 1737 1743 20494020 10.1016/j.athoracsur.2010.02.060

[5] Gaissert H A Mathisen D J Grillo H C Vacanti J P Wain J C Tracheobronchial sleeve resection in children and adolescents J Pediatr Surg 1994 29 02 192 197, discussion 197–198 8176590 10.1016/0022-3468(94)90316-6

[6] Erginel B Ozkan B Gun Soysal F Celik A Salman T Toker A Sleeve resection for bronchial carcinoid tumour in two children under six years old World J Surg Oncol 2016 14 108 27080124 10.1186/s12957-016-0870-0 PMC4832545

[7] Pan W Du L Wu Y Cai W Successful sleeve lobectomy of inflammatory myofibroblastic tumor in a 4-year-old child Ann Thorac Cardiovasc Surg 2014 20(Suppl):430 433 23445794 10.5761/atcs.cr.12.02143

[8] Bölükbas S Baldes N Bergmann T Eberlein M Beqiri S Standard and extended sleeve resections of the tracheobronchial tree J Thorac Dis 2020 12 10 6163 6172 33209454 10.21037/jtd.2020.02.65 PMC7656394

